# Retinitis pigmentosa

**DOI:** 10.1186/1750-1172-1-40

**Published:** 2006-10-11

**Authors:** Christian Hamel

**Affiliations:** 1 Inserm U. 583, Physiopathologie et thérapie des déficits sensoriels et moteurs, Institut des Neurosciences de Montpellier, Hôpital Saint-Eloi, BP 74103, 80 av. Augustin Fliche, 34091 Montpellier Cedex 05, France

## Abstract

Retinitis pigmentosa (RP) is an inherited retinal dystrophy caused by the loss of photoreceptors and characterized by retinal pigment deposits visible on fundus examination. Prevalence of non syndromic RP is approximately 1/4,000. The most common form of RP is a rod-cone dystrophy, in which the first symptom is night blindness, followed by the progressive loss in the peripheral visual field in daylight, and eventually leading to blindness after several decades. Some extreme cases may have a rapid evolution over two decades or a slow progression that never leads to blindness. In some cases, the clinical presentation is a cone-rod dystrophy, in which the decrease in visual acuity predominates over the visual field loss. RP is usually non syndromic but there are also many syndromic forms, the most frequent being Usher syndrome. To date, 45 causative genes/loci have been identified in non syndromic RP (for the autosomal dominant, autosomal recessive, X-linked, and digenic forms). Clinical diagnosis is based on the presence of night blindness and peripheral visual field defects, lesions in the fundus, hypovolted electroretinogram traces, and progressive worsening of these signs. Molecular diagnosis can be made for some genes, but is not usually performed due to the tremendous genetic heterogeneity of the disease. Genetic counseling is always advised. Currently, there is no therapy that stops the evolution of the disease or restores the vision, so the visual prognosis is poor. The therapeutic approach is restricted to slowing down the degenerative process by sunlight protection and vitaminotherapy, treating the complications (cataract and macular edema), and helping patients to cope with the social and psychological impact of blindness. However, new therapeutic strategies are emerging from intensive research (gene therapy, neuroprotection, retinal prosthesis).

## Disease name

Retinitis pigmentosa

## Definition

Retinitis pigmentosa (RP) belongs to the group of pigmentary retinopathies, a generic name that covers all retinal dystrophies presented with a loss of photoreceptors and retinal pigment deposits. RP is a retinal degenerative disease characterized by pigment deposits predominant in the peripheral retina and by a relative sparing of the central retina. In most of the cases of RP, there is a primary degeneration of the photoreceptor rods, with secondary degeneration of cones. Thus, the typical RP is also described as a rod-cone dystrophy, photoreceptor rods being more affected than cones. This sequence of photoreceptor involvement explains why patients initially present with night blindness, and only in the later life would suffer visual impairment in diurnal conditions.

## Diagnostic criteria

### Functional signs

• Night blindness (nyctalopia) is the earliest symptom

• Photophobia appears later

• The visual acuity is preserved in early and mid stages

### Visual field

• Patchy losses of peripheral vision evolving to

• Ring shape scotoma, and eventually

• Tunnel vision

### Fundus

• Pigmentary deposits resembling bone spicules, initially in peripheral retina

• Attenuation of the retinal vessels

• Waxy pallor of the optic disc

• Various degrees of retinal atrophy

### Electroretinogram

• Dramatic diminution in a- and b-wave's amplitudes

• Scotopic system (rods) predominates over photopic (cones) system

## Epidemiology

Prevalence of *non syndromic *RP is approximately 1/4,000 [1-5].

## Clinical description

### Non syndromic retinitis pigmentosa

#### Typical form

RP is a long lasting disease that usually evolves over several decades. However, there are extreme cases with a rapid evolution over two decades or a slow progression that never leads to blindness. The disease course can be conveniently divided into three stages.

**Figure 1:**
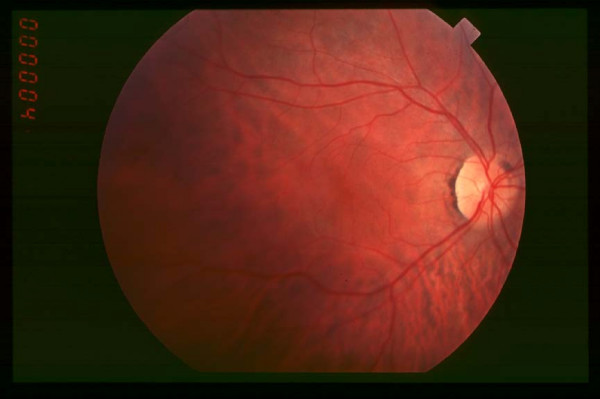
Fundus of patient with retinitis pigmentosa, early stage.

**In the early stage**, night blindness is the main symptom. It may be present from the first years of life or may appear during the second decade, or even later. Mild night blindness is often ignored by the patients and becomes apparent in the teen age, at evening parties. At this stage, there may be peripheral visual field defects in dim light. However, these defects do not exist or are minimal in day light, thus patients have normal life habits and the disease may appear stable. Diagnosis is difficult to establish at this stage, particularly when there is no familial history (about half of the cases). Visual acuity is normal or subnormal. Fundus examination (Figure 1) may seem normal, as bone spicule-shaped pigment deposits are not present or rare. Morevover, the attenuation of retinal arterioles is modest and the optic disc is normal. The visual field test reveals scotomas only in scotopic conditions, while the test is usually done in mesopic conditions. Color vision is normal. The electroretinogram (ERG) is the key test. In most cases, it shows a decreased amplitude of the b-wave that predominates in scotopic conditions. However, ERG may appear normal when the retina is only partially affected, though the decrease in maximum ERG amplitude.

**Figure 2:**
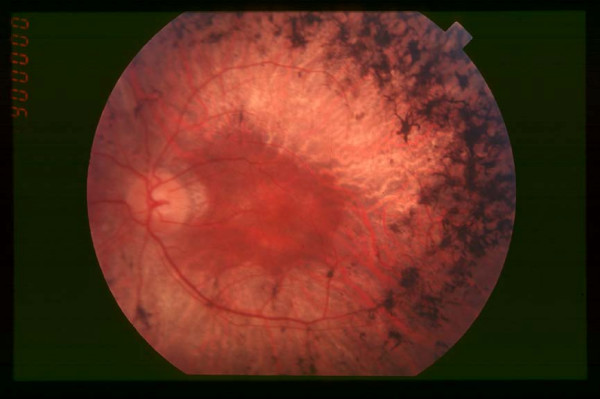
Fundus of patient with retinitis pigmentosa, mid stage (Bone spicule-shaped pigment deposits are present in the mid periphery along with retinal atrophy, while the macula is preserved although with a peripheral ring of depigmentation. Retinal vessels are attenuated.)

**In the mid stage**, the clinical picture is complete. Night blindness is obvious, with difficulties to drive during the night, and to walk at evening and in dark staircases. Patients become aware of the loss in the peripheral visual field in day light conditions through stereotypic situations: while driving, they do not see pedestrians or side-coming cars, they miss hands in handshaking and frequently step into various objects. Consequently, patients adapt themselves by avoiding night driving and circulation in unfamiliar places. Dyschromatopsia to pale colors (particularly blue and yellows hues) is often present. In addition, patients become photophobic, especially in presence of diffuse light (white cloudy weather). This leads to reading difficulties, with a narrow window between insufficient and too bright light. Difficulties with reading are due also to decreased visual acuity, partly because of macular involvement (macular edema or mild foveomacular atrophy) and subcortical posterior cataract. Fundus examination (Figure 2) reveals the presence of bone spicule-shaped pigment deposits in the mid periphery, along with atrophy of the retina. Narrowing of the retinal vessels is evident and the optic disc is moderately pale. In contrast, the extreme periphery and the macular region appear relatively spared, although mild macular involvement is frequent. The ERG is usually unrecordable in scotopic conditions (rods) and the cone responses (30-Hz flickers, bright light) are markedly hypovolted. Phenotypic features of the disease should be carefully registered to guide towards mutation searches. At this stage, evaluation of the rate of the disease progression, based on several year-to-year examinations (visual acuity, ERG and most importantly visual field testing), is mandatory. Indeed, visual field testing shows mild periphery scotomas that tend to enlarge towards extreme periphery and macular area. Cataract, which usually blurs the optic center, should be removed even when there is macular involvement.

**Figure 3:**
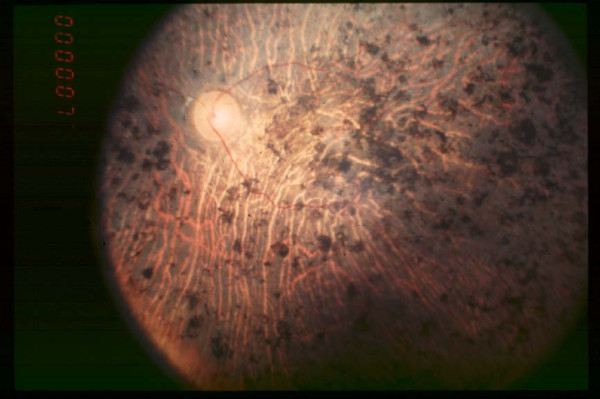
Fundus of patient with retinitis pigmentosa, end stage (Pigment deposits are present all over the retina. Retinal vessels are very thin and optic disc is pale.)

**In the end stage**, patients can no longer move autonomously, as a result of peripheral vision loss (classical tunnel vision), with few degrees of remaining visual field around the fixation point. Reading is difficult and magnifying glasses are necessary. Photophobia is intense. Fundus examination (Figure 3) reveals widespread pigment deposits reaching the macular area. Vessels are thin and the optic disc has a waxy pallor. Fluorescein angiography detects chorioretinal atrophy in the periphery and also in the foveomacular area. The ERG is unrecordable. Even at this stage, the disease progression remains slow, with patients being able to read short passages for years, while being totally incapable to move. However, reading becomes impossible when the central visual field vanishes. Usually, patients continue to perceive light, often in the peripheral visual field.

#### Clinical forms

There are many forms of non syndromic RP that can be classified on the basis of several criteria:

##### • Age of onset

Early onset RP is diagnosed when symptoms of mid stage RP are already present at the age of two years. These forms may be difficult to distinguish from Leber's congenital amaurosis (LCA) in which a severe visual impairment is present from birth or at least in the first year of life. In fact, mutations in *RPE65*, *CRB1*, *CRX *and *TULP1 *genes cause retinal dystrophies that are diagnosed as either LCA or RP, depending on the age of onset [6].

Alternatively, late onset RP is diagnosed when symptoms of early stage RP are apparently beginning at or after mid life. One possibility is that moderate night blindness from infancy is ignored by the patient and slowly worsens to the point where it becomes clinically apparent. Another possibility is RP truly to begin late. In this case, one must search for a non genetic cause of similar phenotype such as ocular trauma or inflammation/infection, paraneoplasic syndromes, association with a spinocerebellar ataxia, particularly if there is a rapid evolution of the symptoms.

#### Fundus appearance

• Absence or scarcity of pigment deposits may occur. This is frequent in myopia because of the retinal pigment atrophy linked to this condition. In other cases, the amount of pigment deposits may vary and does not necessarily reflect the severity of the disease.

• Localization of the lesions. There are regional or sectorial forms in which only one or two quadrants are affected (*RHO*, *PRPF31 *mutations). The lesions may also be localized as a ring around the macula (pericentral), the optic disc (parapapillary) or predominantly along retinal veins (paraveinous). In some cases, there is paraarteriolar retinal pigment epithelium preservation (*CRB1 *mutations). Finally, there are rare cases of unilateral RP for which a local cause (trauma) should be actively searched.

• Other lesions. White dots or whitish spots can be present as in retinitis punctata albescens (*RLBP1 *mutations). Macular atrophy can be quite prominent from the mid stage of the disease (*RDS *and *CRX *mutations).

#### Mode of inheritance

• Autosomal dominant forms are usually the mildest forms, with some cases starting after the age of 50, although severe disease can also appear. Variations in penetrance are frequent, particularly in case of *PAP1*, *PRPF31 *and *RP1 *mutations. In genetic counseling, one should always suspect autosomal dominance in apparently sporadic mild cases, especially when ascendants have not been thoroughly examined or are unknown.

• Autosomal recessive forms start typically during the first decade, although some mild forms can be encountered.

• X-linked forms also start early and are frequently associated with myopia. Although transmission is recessive in most cases, there are some families in which dominant inheritance with affected females is found.

• Digenic form: rare cases have been described in which heterozygous mutations in *ROM1 *in combination with heterozygous mutations in *RDS *cause digenic RP. These forms are inherited in a pseudo-dominant pattern (1/4 risk).

### Syndromic retinitis pigmentosa

Many syndromes associate with various types of pigmentary retinopathies.

#### Frequent syndromes

• **Usher syndrome **is the most frequent syndromic form in which typical RP is associated with neurosensory deafness. About 14% of all RP cases are, in fact, Usher syndrome [7]. Deafness, generally congenital and stable, may be profound (type 1) or moderate/medium (type 2). In some cases deafness occurs during the first decade and worsens progressively (type 3). Mutations in at least 11 genes are responsible for Usher syndrome (for review see [8]).

• **Bardet Biedl syndrome (BBS) **is less frequent than Usher syndrome (prevalence 1/150,000 [9]). The phenotype is characteristic and associates RP (often of cone-rod dystrophy type) with obesity already present in childhood, mental retardation or mild psychomotor delay, post axial polydactyly, hypogenitalism and renal abnormalities that lead to renal failure. BBS is due to mutations in at least 11 genes [10,11], with cases of triallelic digenic inheritance [12]. The rare Alstrôm syndrome (due to *ALMS1 *gene mutation) resembles BBS and presents with deafness, diabetes mellitus and acanthosis nigricans.

#### Less frequent syndromes

##### Renal abnormalities

• **Senior Loken syndrome (SLS) **associates an usually severe RP (sometimes diagnosed as LCA) with nephronophtisis (renal atrophy frequently evolving towards renal failure requiring transplantation), or sometimes a milder RP that is discovered later in life. At least four genes (*NPHP1, NPHP3-5*) encoding nephrocystins are involved in this disease [13].

• **Alport syndrome: **deafness and progressive nephritis are associated with yellow flecks around the macula, rather than with an authentic RP.

##### Dysmorphic syndromes

• **Cohen syndrome **associates RP to a particular facial dysmorphism (prominent upper incisors) with short stature, mental retardation, long and narrow hands, and neutropenia. One causative gene (*COH1*) that encodes a protein involved in vesicular trafficking, is related to this syndrome [14].

• **Jeune syndrome **associates RP with a thoracic hypoplasia, brachydactyly and chronic nephritis. One locus has been identified (asphyxiating thoracic dystrophy, *ATD*).

• **Cockayne syndrome **is characterized by dwarfism, progeria, mental retardation, and retinopathy with fine granular spots.

##### Metabolic diseases

• **Methylmalonic aciduria with homocystinuria **is caused by genetic defects in enzymes that metabolize vitamin B12. Rare cases present with macular atrophy, salt-and-pepper retinopathy, and vascular attenuation.

• **Abetalipoproteinemia (Bassen Korntzweig disease) **is characterized by progressive ataxia, steatorrhea, reduction of plasma lipids and pigmentary retinopathy that resembles retinitis punctata albescens in some cases.

• **Bietti's disease **shows characteristic microcrystalline deposits in fundus and cornea. Patients undergo progressive RP evolving towards chorioretinal atrophy. The causative gene, encoding a form of cytochrome P450 (*CYP4V2*), has been recently discovered [15].

• **Cystinosis **presents with typical crystal deposits in the cornea and pigmentary retinopathy in a highly photophobic patients with short stature. Accumulation of cystine in other body parts leads to hypothyroidism, diabetes mellitus, and renal failure. The causative gene (*CTNS*) encodes a protein (cystinosin) involved in the lysosomal transmembrane transport of cystein [16].

• **Mucopolysaccharidoses **are characterized by facial and bony changes, mental retardation and corneal clouding. Only types I, II and III show pigmentary retinopathy.

• **Zellweger (cerebro-hepato-renal) syndrome.**

• **Hyperoxaluria type I with retinal atrophy in spots.**

• **Neonatal adrenoleukodystrophy with leopard spots in fundus.**

• **Infantile Refsum disease **(caused by mutation in the *PEX1*, *PEX2 *or *PEX26 *genes) presents with elevated phytanic acid and pigmentary retinopathy with characteristic prominent macular involvement.

• **Adult Refsum disease **caused by mutation in the gene encoding phytanoyl-CoA hydroxylase (*PAHX *or *PHYH*) or the gene encoding peroxin-7 (*PEX7*) presents with highly elevated phytanic acid, anosmia, deafness, and RP.

• **Peroxisomal disorders other than Refsum disease**: except for the rhizomelic chondrodysplasia punctata, all children with disorders of peroxisomal assembly who survive long enough develop pigmentary retinopathy.

##### Neurological diseases

• **Neuronal ceroid lipofuscinosis **(also called Batten disease or amaurotic idiocies), associates mental retardation, seizures, ataxia and retinal degeneration. The retinal disease starts with macular involvement (red-cherry spot) and later spreads to peripheral retina. The protein, encoded by *CLN3*, is found in the lysosomes and in synapses [17].

• **Joubert syndrome **(JBTS) is a phenotypically heterogenous syndrome that associates various central nervous system (CNS) developmental abnormalities including the so-called "molar tooth sign", cerebellar vermis hypoplasia and cerebral cortex defects, with renal cysts, and pigmentary retinopathy. There are overlaps with Senior Loken syndrome, as *NPHP1 *is a causative factor in about 2% of JBTS4. Another causative gene, *AHI1*, has been recently discovered in the JBTS3 form [18,19]. There are two other loci (JBTS1-2).

• **Autosomal dominant cerebellar ataxia type II (SCA7) **shows a retinal disease, which often begins with a granular macula and then spreads out to the whole retina. It is due to trinucleotide expansions in the transcription factor ataxin-7 and anticipation effect is found [20].

• **Myotonic dystrophy shows cataract and sometimes pigmentary retinopathy.**

• **Hallervorden-Spatz syndrome **shows progressive dysarthria and dementia, iron deposition, and flecked type retinopathy with bull's eye maculopathy.

## Etiology

### Causative genes

#### Non syndromic

RP are genetic disorders inherited as mendelian traits in most cases. Except for mutation in a few genes that can cause both autosomal dominant and recessive forms of RP (*NRL, RP1 *and, exceptionally, *RHO*), most genes involved in the disease are linked to only one form of inheritance. There are also some rare RP cases due to mitochondrial DNA mutations [21] and to digenic diallelic inheritance involving *RDS *and *ROM1 *genes [22]. Uniparental isodisomy and incomplete penetrance have also been described (reviewed in [23]).

In 1990, the first gene involved in RP, *Rhodopsin*, has been identified [24]. It encodes the rod visual pigment. Since then, it has been established that mutations in many genes may cause RP [25]. To date, 45 known genes/loci have been identified in *non syndromic *RP, including 15 for autosomal dominant- (14 cloned, one mapped), 24 for autosomal recessive- (18 cloned, six mapped), five for X-linked- inheritance (two cloned, three mapped), and one, *ROM1*, which has been found mutated only in digenism with *RDS*. It has been estimated that the cloned genes account for about 50% of dominant RP, 40% of recessive RP and approximately 80% of X-linked RP, indicating that many genes remain to be identified [26].

The gene products localize in rods (sometimes in rods and cones), being involved in various metabolic pathways. They include proteins of the rod visual transduction (rhodopsin, **α **and **β **subunits of the rod phosphodiesterase, **α **and **β **subunits of the rod cGMP gated channel, arrestin, guanylate cyclase activating protein 1B), cytoskeleton proteins (peripherin/RDS, ROM1, fascin 2), proteins presumably involved in trafficking (RPGR, RP1, RP2, prominin-like 1), in photoreceptor differentiation (NRL, NR2E3, CRX), in mRNA splicing (PRPC8, HPRP3, PRPF31, PAP1), in the composition of extracellular matrices (USH2A), and in lipid (ABCA4, CERKL), nucleotide (IMPDH1) or other (TULP1, CRB1, MITS2, CA4, SEMA4A) metabolic pathways. In addition, RP is also caused by mutations of genes expressed in the photoreceptor supporting tissue, *i.e*. the retinal pigment epithelium (*RPE*), the encoded proteins being involved in the retinol metabolism (the retinol isomerase RPE65, the 11-cis retinoid transporter CRALBP, the lecithin retinol acyl transferase LRAT, RGR) or in the phagocytosis of the photoreceptor outer segments (cMERTK).

### Specificities of photoreceptors

The genetic heterogeneity of RP is difficult to correlate with the fairly homogeneous phenotype of the disease. Photoreceptors, and particularly rods, may require a highly regulated environment to function properly and any alteration of this environment may render these cells prone to apoptosis, causing loss of rods and cones. Rods have a very elongated outer segment that contains several hundreds of membrane discs in which visual transduction occurs. Discs contain huge amounts of visual transduction proteins, particularly rhodopsin (~4 × 10^7 ^molecules per rod) and cytoskeleton proteins. Discs of the apex of the rod outer segment are phagocytosed daily by the retinal pigment epithelium (RPE), and this phenomenon is compensated by a daily boost of disc synthesis at the base of the outer segment. This requires an intense activity of mRNA and protein synthesis, as well as an important protein trafficking from the rod inner segment, through the connecting cilium, to the rod outer segment. This cellular activity generates an important energy consumption, requiring high content of mitochondria and oxygen, and mechanisms to protect the cell against the oxidative stress.

### Possible common pathways to photoreceptor cell death

Loss of the rod outer segment may be caused by mutations that lead to its destabilization (mutations in cytoskeleton or trafficking proteins). This would considerably shorten the photoreceptor layer and expose the photoreceptor cell body to high pressure levels in oxygen, hence oxygen toxicity. Mutations that lead to diminution in the ability to respond to high demand of energy or mRNA/protein synthesis may somewhat destabilize the outer segment. Other mechanisms that may be involved are calcium toxicity or metabolic exhaustion by permanent opening of the cGMP-gated channel, due to defective visual transduction proteins, or, conversely, due to low calcium when visual transduction permanently activated [27]. Finally, alterations in critical RPE functions, such as disc phagocytosis or retinol metabolism, may also deregulate the fine balance of photoreceptor metabolism.

### Research process

Identification of the causative genes is a necessary step towards the understanding of RP pathophysiology. With the use of genetic databases, it can be reasonably assumed that most genes responsible for autosomal dominant and X-linked RP will be known within the next few years. This will not be as straightforward in autosomal recessive RP and some sporadic cases, as these forms seem to be associated with an extreme genetic diversity. For each gene, we then need to explore what causes the decrease in the visual performance on the one hand, and how the mutated or absent protein causes the loss in photoreceptor, on the other hand. This implies the development of animal models and long lasting experiments based on cell and molecular biology techniques. From this knowledge, therapeutic trials are being conducted.

## Diagnostic methods

Clinical diagnosis is based on the presence of night blindness and peripheral visual field defects, lesions in fundus, hypovolted ERG traces, and progressive worsening of these signs. Full field ERG is the key test, particularly when patients are asymptomatic and show normal fundus at early stages of the disease or in autosomal dominant forms with variable penetrance, since it is usually hypovolted before the appearance of clinical signs (night blindness). It is important to ascertain the diagnosis by repeating the examination one or two years after it has been first established. Multifocal ERG and electrooculogram are not essential to establish the diagnosis.

At present, a systematic molecular diagnosis is not routinely performed, due to the tremendous genetic heterogeneity of the disease. However, rapid and large-scale mutation screening techniques are developing and several laboratories perform search for mutations in the most frequently involved genes, including:

• *RPGR *that accounts for at least 10% of all cases of non syndromic RP, including 55% of X-linked RP and until 25% of sporadic RP. It is also involved in cases of X-linked cone or macular dystrophies.

• *RHO *that accounts for 15 to 20% of dominant non syndromic RP cases.

• *USH2A *may account for 1/3 to 1/2 of cases with Usher syndrome and may be involved in at least 16% of cases with recessive non syndromic RP [28].

In some instances, molecular diagnosis for certain genes is performed by the laboratories that have discovered them.

The currently known genes responsible for RP account for 50–60% of the cases, and strategies to test in a short time several dozen of genes for a single patient DNA are emerging [29,30].

## Differential diagnosis

Leber's congenital amaurosis (LCA), which also belongs to the group of pigmentary retinopathies, must be differentiated from RP, although some genes are involved in both LCA and RP. RP is also clearly different from the macular dystrophies in which the extent of the lesions are limited to the macula. Cone dystrophies, due to cone degeneration while rods remain unaffected or only moderately affected, must also be excluded, although some genes cause either cone dystrophies or RP. Finally, cone-rod dystrophies that are usually viewed as a subclass of RP should be distinguished from typical RP (rod-cone dystrophy).

Various entities resemble RP:

### Night blindness in non degenerative diseases

In these cases, in contrast to RP, the disease is not evolving with time.

• Congenital stationary night blindness. In autosomal forms, symptoms are limited to night blindness, while X-linked forms are associated with a limited visual acuity.

• Fundus albipunctatus is a rare condition in which fine, white deposits are visible in fundus. The fundus aspect is similar to retinitis punctata albescens (see above), but there is usually no signs of degeneration (narrowing of retinal vessels, retinal atrophy), although some cases may undergo macular degeneration [31].

• Vitamin A deprivation syndrome mimics the signs of RP with night blindness and is associated with keratitis. If vitamin A supplementation is given early, the symptoms disappear but after a certain point the lesions become irreversible.

### Non evolving pigmentary retinopathies

The aspect of the fundus is often that of salt-and-pepper pigmentary retinopathy or deposits of pigment with various shape, often dot-like.

• Congenital infections like rubella (salt-and-pepper retinopathy) or syphilis (pseudo-retinitis pigmentosa or leopard skin retinopathy).

• Carriers of X-linked disorders like choroideremia, ocular albinism, RP. This helps to recognize carriers, in particular for RP in which a yellowish reflex may be present in fundus.

• Mitochondrial diseases like Kearns-Sayre syndrome (ophthalmoplegia), although there may be progressive degeneration of photoreceptors.

• Grouped congenital hypertrophy of the retinal pigment epithelium with characteristic bear-like footprints in fundus.

### Choroidal dystrophies

In all cases there is a marked atrophy of the choriocapillaris that is readily diagnosed by the absence of fluorescence in fluorescein angiography.

• Choroideremia, an X-linked disorder, due to mutations in *CHM *encoding the Rab Escort Protein 1 (REP1) and accounting for about 2% of pigmentary retinopathies.

• Gyrate atrophy, a very rare autosomal recessive disorder, due to deficiency in ornithine aminotransferase.

### Vitreoretinopathies

In these conditions, the vitreous and inner layers of the retina are also affected. Retinal detachment and retinal vasculopathy are often present.

• Retinoschisis, in most cases the juvenile X-linked retinoschisis with typical spoke-wheel-like lesions in the fovea, is due to mutations in *XLRS1 *encoding a protein involved in the adhesion of retinal cells. End stage X-linked retinoschisis are difficult to distinguish from RP because of the macular degeneration and frequent pigmented lesions in peripheral retina. There is also the autosomal recessive Goldman Favre syndrome in which patients have night blindness from infancy and show foveal retinoschisis in fundus. It is the same disease as the Enhanced S-Cone Syndrome (ESCS) due to mutations in *NR2E3*, that presents with characteristic whitish and secondarily round pigmented lesions in retinal periphery when evolved.

• Hereditary vitreoretinopathies, the most frequent ones being several autosomal dominant conditions: familial exudative vitreoretinopathy, Wagner disease and Stickler syndrome.

• Inflammatory diseases of the eye, birdshot choroidoretinopathy, serpiginous retinopathy, multifocal placoid pigment epitheliopathy, sarcoidosis. The presentation and fundus are clearly different from RP but there may be a secondary degeneration mimicking RP.

### Maculopathies

Large, extended maculopathies may be difficult to differentiate from end stage RP.

• Stargardt disease, due to mutations in *ABCA4*. Null mutations in this gene can also be responsible for authentic RP.

• Cone dystrophies, in some cases presenting with a minimal rod involvement.

• Sorsby's disease, in extended cases.

### Secondary pigmentary changes

Several diseases may lead to secondary RP with variable disease course.

• Intoxication with various drugs including thioridazine and chloroquine. Although chloroquine usually leads to "bull's eye maculopathy", there are some cases of RP-like pigmentary retinopathies that may continue to progress even after discontinuation of the drug intake.

• Inflammation (pars planitis, Behcet disease, sarcoidosis, subacute diffuse unilateral neuroretinitis) may rarely be complicated with RP.

• Sequelae of severe gravidic toxemia, uveal effusion syndrome or trauma.

• Parasitic infections such as onchocercosis.

## Genetic counseling

Once the diagnosis is made, patients should be informed and familial surveys recommended. Genetic counseling is always advised since all genetic forms can be encountered in RP. A precise phenotypic diagnosis is always mandatory and is particularly useful in the absence of familial history or in sporadic cases.

## Antenatal diagnosis

Prenatal diagnosis (amniocentesis or chorionic biopsy) raises an ethical issue: whether the investigative risks associated with these invasive prenatal procedures are justified in a non life-threatening disease is questionable. Prenatal diagnosis can be performed in families in which the responsible gene has been identified, particularly in families with early onset and severe RP.

## Management including treatment

Currently, there is no therapy that stops the evolution of pigmentary retinopathies or restores the vision. However, there are several therapeutic strategies aimed at slowing down the degenerating process, treating the complications and helping patients to cope with the social and psychological impact of blindness.

### Slowing down the degenerating process

#### Light protection

Clinical evidence and data from animal studies indicate that some genetic types of pigmentary retinopathies are partly light-dependent [32]. Thus, patients with pigmentary retinopathies are recommended to wear dark glasses outdoor. Wearing of yellow-orange spectacles minimizes photophobia. Eyeshade and lateral protection help to protect against dazzling side-coming light rays.

#### Vitaminotherapy

Vitamins A and E may protect the photoreceptors by trophic and anti-oxidant effects, respectively. Previous studies have demonstrated that long term (5–12 years) vitamin A supplementation at doses of 15,000 units per day slightly slowed down the loss in ERG amplitude, while vitamin E at 400 units per day had adverse effects [5]. However, the conclusions of this study were debated [33], thus there is no consensus about the usefulness of vitamin A treatment. If vitamin A supplementation is proposed, levels of serum retinol (normal <3.49 **μ**mol/l, *i.e*. <1 mg/l) and triglyceridemia (normal <2.13 mmol/l, *i.e*. <0.19 g/l) should regularly be checked, as well as liver enzymes (aspartate aminotransferase, alanine aminotransferase and alkaline phosphatase) since vitamin A storage occurs mainly in this organ. Vitamin A should not be given to RP patients with mutations in *ABCA4*. In a recent study, patients were given docosahexaenoic acid (DHA) supplementation at 1200 mg/day, in addition to vitamin A. It was shown that the course of the disease was initially slowed down by the addition of DHA, but this beneficial effect did not last over 2 years [34].

### Treatments of complications

The most frequent complications are cataract and macular edema.

#### Cataract

It is a posterior central subcapsular cataract with a clear nucleus, which is usually present at mid stage in the evolution of the disease. Although the cataract is not widespread, its central position blurs the remaining central visual field. Therefore, cataract provokes a sight restriction and generates photophobia. Phacoemulsification with implantation of intraocular lens is thus required.

#### Macular edema

Macular edema occurs frequently, causing a decrease in the visual acuity. Acute episodes of macular edema may be successfully treated with carboanhydrase inhibitors such as acetazolamide sodium at a daily dose of 500 mg or less. However, the macular edema in RP patients is most often chronic and does not improve with this treatment [35]. Topical administration of dorzolamide is inefficient [36].

#### Inflammatory reactions

Mild inflammatory reactions occur frequently in the vitreous and are often associated with macular edema, vascular diffusion visible on fluorescein angiogram, and early cataract. Although these reactions do not require a specific treatment, some cases present with large exsudates in the peripheral retina (pseudo Coats) that leads to retinal detachment and rapid evolution towards blindness. This latter complication has been found recurrently in RP linked to *CRB1 *mutations [37]. Cryotherapy or laser treatment are required for resorption of the exudates.

#### Others

Myopia associated with X-linked RP requires management and routine examinations as for non RP patients. Glaucoma is not associated with RP but the presence of increased intraocular pressure in RP patients should be cautiously checked in order to prevent more rapid deterioration of the visual field.

### Management of blind patients

Psychological help is often necessary at milestones in the course of the disease: announcement, occurrence of moving difficulties and loss of reading. This support can be provided by either professionals or supportive patients' associations. Patients should be oriented towards institutions that help them to rehabilitate (short- and medium-stay stages and others [38] and to obtain new professional skills.

### Future treatments

Enormous efforts have been invested to identify the involved genes, to unravel the underlying pathophysiological mechanisms, and to find efficient treatments. Search for new therapies follows several strategies, which may be non exclusive. None of these future treatments is currently operating in humans.

#### Treating the cause of the disease

##### • Gene therapy

This approach requires the implicated genes to be identified and therefore, the availability of efficient genotyping methods. The strategy is relatively simple for RP due to loss-of-function (usually recessively inherited). In this case, one expect that the expression of the wild-type cDNA in the appropriate cell (photoreceptor or RPE) will avoid cell death. However, it is more complicated for RP due to dominant negative pathogenic mechanisms in which the expression of the mutated gene should be inhibited, by use of ribozymes or siRNA for example. In the last 10 years, studies have been carried out in several animal models. Although all showed a significant rescue of photoreceptors, there was still progressing photoreceptor cell death, which could be due to an inappropriate expression level of the therapeutic gene and to an insufficient percentage of transduced photoreceptors. The most advanced studies have been performed for LCA in a large animal model (the Briard dog) in which the surgical administration in the subretinal space of AAV vectors carrying the RPE65 cDNA allowed to restore vision in four month-old dogs in USA [39,40] and in France [41]. Five years later, the dog vision seems stable, although the very long-term efficiency still remained to be ascertained. Promising results have been obtained in a mouse model of X-linked retinoschisis [42]. It is expected human trials in RPE65 patients to be carried out soon in the USA, UK, France, and other countries.

##### • Pharmacological treatment

In those cases where some aspects of the pathophysiological mechanism are known, pharmacological treatment may be a good choice, as it offers the advantages of using available drugs with known toxicity that can be modulated. Pharmacological agents can compensate for a biochemical defect, and enhance or inhibit the activity of various effectors. Calcium-channel blockers have been tried in several animal models of RP [43], yet with limited success [44]. Another example is Stargardt disease in which the use of visual cycle inhibitors has been shown to slow down the toxic accumulation of lipofuscin in the RPE in a mouse model [45,46]. Supply of 9-cis retinal has been shown to restore the rod activity in a *Rpe65-/- *mouse model of LCA [45]. NAD analogues supply in RP due to *IMPDH1 *defects may also be efficient [48]. It might be speculated that the alternative of pharmacological treatments would be explored in more details in the future, as the mechanisms of the various forms of RP will be progressively unraveled.

#### Coping with photoreceptor cell death

A general problem with the treatment of the primary cause of the disease is that beyond certain stage in the evolution, non-cell autonomous mechanisms leading to cell death may overwhelm the potential benefits of gene- or pharmacological therapies. Cell death may be due to the release of proapoptotic signals in the photoreceptor environment, or to the lack of survival factors normally produced by the living cells. The latter has been confirmed by the discovery that rods produce factors that are necessary for cone survival [49]. Thus, in typical RP, rods die because they express a mutated gene, and cones, which do not express the mutated gene, are secondarily degenerating because of the lack of rod factors. Therefore, the supply of rod factors in the retina would protect cones against secondary degeneration.

##### • Neuroprotection using growth factors

Several growth factors, including ciliary neurotrophic factor (CNTF), glial-derived neurotrophic factor (GDNF), cardiotrophin-1, brain-derived neurotrophic factor (BDNF) and basic fibroblast growth factor (bFGF) have some efficacy in animal models, that varies from one model to another. Their short half-life requires their delivery *in situ*. Since iterative intravitreal injections are not recommended, several strategies like use of encapsulated cells producing bFGF placed in the vitreous cavity [50] and gene transfer of GDNF in resident cells [51] have been tried. These factors, however, have side effects including retinal neovascularization and cataract. For example, CNTF allows an excellent preservation of retinal integrity in several animal models, but it causes a decrease in the ERG response of the retina by yet unknown toxic mechanism [52]. Nevertheless, encapsulated cells releasing CNTF of vitreous of patients with RP is currently under Phase I clinical investigation [53].

##### • Neuroprotection using antiapoptotic factors

In animal models, gene transfer of anti-apoptotic *bcl-2 *slows down the photoreceptor cell death [54] as well as the use of an inhibiting peptide of caspase-3 [55].

##### • Rod-derived cone viability factor

Léveillard *et al*. [56] identified a rod-derived cone viability factor (RdCVF) that appears to be a truncated thioredoxin-like protein which significantly delays cone death in the *rd1 *mouse model of RP. Studies are ongoing to test whether this factor will be efficient in other forms of RP.

#### Restoration of visual function

Beside therapies aimed at preserving visual function and preventing cell death, one would like to find out ways of restoring the visual function. This is a tremendous challenge since (as a general rule for neurons in the CNS) human photoreceptors are not produced and do not divide after birth, therefore, their loss is irreversible. In addition, the loss of photoreceptors leads to a dramatic remodeling of the retinal circuits which would probably modify the visual information process if correct implantation of new photoreceptors was possible. Nevertheless, numerous teams are now working to achieve visual restoration either by photoreceptor replacement or by means of artificial devices.

##### • Cell or tissue transplantation

Experiments have been tried to transplant retinal cells from fetuses or adult retina in humans, and layers of photoreceptors or even entire retina in animals models (rats and rabbits). Generally, the survival of transplanted photoreceptors is readily observed, but they do not properly organize in the retina (forming rosettes) and lack, with rare exceptions, functional synapses. Researchers are also becoming interested by using stem cells, embryonic or adult, from retina or from other tissues. Although very interesting to study, these therapeutic approaches are still far from realistic use in a near future.

Conversely to photoreceptors, it has been proven that the RPE grafts rescue the photoreceptors in Royal College of Surgeons (RCS) rat model, in which a mutation in *c.Mertk *causes a retinal dystrophy by lack of outer segment phagocytosis of the RPE, and in rare cases of RP in humans [57,58]. In RP due to RPE defects, RPE transplantation is then theoretically possible, but one has to resolve the immunogenic reaction against allogenic, wild type RPE.

##### • Retinal prosthesis

Microphotodiodes arrays that replace degenerated photoreceptors or more sophisticated devices that capture light and stimulate the retina, optic nerve or visual cortex have been developed. Several clinical trials have essentially demonstrated the tolerance of the implanted devices. Today, they represent the basis for further studies towards improvement of the future devices resolution.

## Prognosis

Evaluation of the prognosis is not an easy task as the quality of vision is depending on several features such as peripheral visual field, visual acuity and perception of contrasts which may not change in concert. For example, one patient with a long-evolved RP may feel fortunate because of a relatively good visual acuity to 4/10, even if his/her tiny visual field limited to 5° around the fixation point does not allow autonomous walking outside, while a younger patient, with a better visual field will feel unfortunate with a visual acuity less than 1/10 due to macular atrophy.

Few studies have addressed the question about the disease prognosis, even though this is a very important concern for patients. The rate of decline in visual performance is depending on many parameters that include the gene and type of mutations as well as other genetic and environmental factors. It has been recently established that the disease course in patients with pericentral RP is slower that those with typical RP [59]. There are also several clues, such as the Optical Coherence Tomography (OCT) third high-reflectance band, which may help to predict which patients are more likely to lose visual acuity with the decline of the retinal thickness [60].

Overall, we clearly need to use standardized tests over extended periods of time to precisely determine clinical subgroups who will be relevant for clinical trials, in particular to appreciate the efficacy of treatments.

## Unresolved questions

Cloned genes account for 40 to 54% of the autosomal dominant cases, 61 to 89% of the X-linked cases, and probably less than 1/3 of the autosomal recessive cases, not taking into account all the sporadic cases (45% of all RP cases). Therefore, it can be broadly estimated that half the genes have yet to be discovered. It is anticipated that modifier genes play important roles, in particular in incomplete penetrance of autosomal dominant RP and in sporadic cases. Those modifier genes, that could also be used for therapeutic prospects, remain to be discovered. The understanding of the role of the encoded proteins often requires many years. Today, for a number of proteins, substantial information about their function is available, while some of them remain poorly known.

A challenging issue is the elucidation of the precise steps leading from a gene mutation to photoreceptor degeneration. Data from animal models and clinical studies suggest that photoreceptors die by apoptosis at a linear rate throughout life (named the one-hit hypothesis), implying that they have a given probability to undergo apoptosis that remains constant from early to late stages of the disease [61]. For certain genes or severe mutations, this probability will be high, while for others it will be lower. The results of experimental and clinical studies clearly indicate that the mechanisms of photoreceptor degeneration are multiple. In all genetic forms of RP studied till now, data are incomplete. In addition, it is likely that several apoptotic pathways are involved in the photoreceptor loss, sometimes concurrently, and this also needs to be carefully investigated. This knowledge is crucial to design therapies. The efficacy of various potential treatments has to be proven in large animal models and in humans. For example, gene replacement therapy for RDS in mouse improves photoreceptor ultrastructure, but there is no significant effect on photoreceptor cell loss [62].
